# Metabolic reprogramming in the immune microenvironment of Ewing sarcoma: from mechanisms to therapeutic strategies

**DOI:** 10.3389/fimmu.2026.1962178

**Published:** 2026-10-28

**Authors:** Lingyun Tang, Haoling Zhou, Jia Liu

**Affiliations:** 1 Department of Emergency Medicine, The Second Affiliated Hospital, University of South China, Hengyang, China; 2 Department of Clinical Medicine, Changsha Medical University, Changsha, China; 3 Department of Geriatrics, Shaoyang Central Hospital, Shaoyang, China

**Keywords:** Ewing sarcoma, EWSR1−FLI1, immunometabolism, immunotherapy, lactate, metabolic reprogramming, tryptophan–AhR axis, tumor immune microenvironment

## Abstract

Ewing sarcoma is the second most common primary malignant bone tumor in children and adolescents, with the EWSR1-FLI1 fusion gene serving as its core driver event. Although multi-agent chemotherapy has achieved nearly 70% five-year survival in patients with localized disease, the prognosis for those with metastatic or relapsed/refractory disease remains poor, with long-term survival rates below 30%, and no substantial breakthrough has been made in first-line regimens over the past decade. Immune checkpoint inhibitors have revolutionized the treatment landscape for various adult solid tumors, but their efficacy in Ewing sarcoma—a typical “cold” tumor—is limited, owing to its low tumor mutational burden, sparse neoantigens, and highly immunosuppressive microenvironment. Recent studies suggest that EWSR1-FLI1 not only reshapes transcriptional programs but also functions as a metabolic “master switch, “ driving enhanced glycolysis, altered glutamine and tryptophan metabolism, and lipid metabolic remodeling. These metabolic alterations, in turn, produce lactate, kynurenine, lipid mediators, and local hypoxia, which suppress effector T cells and natural killer cells, induce regulatory T cells, and polarize macrophages toward a protumor phenotype, thereby forming a self-reinforcing causal chain between metabolism and immunity. This review systematically delineates this chain—from fusion-driven metabolic reprogramming, through metabolism-mediated immunosuppressive mechanisms, to intervention strategies targeting the metabolism–immunity interface. We focus on glycolysis inhibition, blockade of the tryptophan–AHR axis, lipid metabolism interventions, as well as novel surface targets (B7-H3, STEAP1, ENPP1, IL1RAP, MIF-CD74) for cell-based therapies and combination strategies of metabolic modulation with immunotherapy. We further analyze the practical obstacles to translating these strategies into clinical practice and, based on a critical synthesis of available evidence, Propose a metabolic–immune coupling model anchored on the active state of EWS-FLI1 to explain plasticity-driven resistance. This model provides a translational roadmap grounded in metabolic subtyping with the goal of “converting cold tumors to hot, “ offering a new framework for improving outcomes in Ewing sarcoma.

## 1 Introduction

Ewing sarcoma is a highly aggressive small round cell sarcoma that predominantly arises in bone and soft tissue, and it is the second most common primary malignant bone tumor in children and adolescents, after osteosarcoma (1–3). Its incidence varies markedly across populations, being significantly higher in individuals of European descent than in East Asian and African populations, suggesting a genetic susceptibility background (3–5). Current standard therapy is based on interval-compressed chemotherapy alternating vincristine/doxorubicin/cyclophosphamide with ifosfamide/etoposide, combined with local surgery and radiotherapy (6, 7). This regimen yields long-term survival rates approaching 70% for patients with localized disease; however, patients who present with metastases at diagnosis or who become relapsed/refractory have very limited responses, and their long-term survival has remained stalled at 20%–30% for decades (8–11). Systemic treatment options for metastatic and relapsed/refractory populations are scarce, and real-world long-term outcomes remain unsatisfactory (12–14).

Part of this therapeutic dilemma stems from the immunologically “cold” nature of Ewing sarcoma. Unlike “hot” tumors such as melanoma and non-small cell lung cancer, Ewing sarcoma exhibits an extremely low tumor mutational burden, sparse neoantigens, insufficient infiltration of cytotoxic T cells and mature dendritic cells, and a predominance of macrophages with an immunosuppressive phenotype (11). Multiple immunoprofiling studies have shown that M2-type macrophages constitute the highest proportion of tumor-infiltrating leukocytes and are associated with worse event-free survival, while immune checkpoint blockade alone rarely elicits durable responses in this disease.

The molecular cornerstone of Ewing sarcoma is the EWSR1-FLI1 fusion gene generated by the chromosomal translocation t(11;22)(q24; q12) (15). Approximately 85% of cases are driven by this fusion, with the remainder harboring fusions of EWSR1 with other ETS family members (16–18). As an aberrant transcription factor, EWSR1-FLI1 binds to GGAA microsatellites in the genome and induces neo-enhancers, extensively rewiring transcriptional programs. In recent years, it has become increasingly recognized that its role extends far beyond transcription: it simultaneously acts as a metabolic “master switch” that, through reprogramming glucose, amino acid, and lipid metabolism, not only meets the biosynthetic demands of the tumor but also actively shapes a microenvironment that is hostile to antitumor immunity (2). This review follows the main axis of “driver gene → metabolic reprogramming → immunosuppression → targeted intervention → obstacles and translation, “ and systematically examines metabolic reprogramming within the immune microenvironment of Ewing sarcoma and the therapeutic opportunities it presents.

## 2 EWSR1-FLI1: the master switch of metabolic reprogramming

### 2.1 Fusion gene, GGAA enhancers, and chromatin remodeling

Through its FLI1 moiety, EWSR1-FLI1 recognizes GGAA motifs and acts as a pioneer factor at GGAA microsatellite repeats, opening closed chromatin and *de novo* establishing tumor-specific enhancers (19–21). The EWSR1 portion contains a low-complexity prion-like domain that recruits the BAF (SWI/SNF) chromatin remodeling complex to these microsatellites, driving transcription of target genes (22, 23). Microsatellite repeat length varies among individuals and populations, thereby influencing enhancer activity and susceptibility, which partially accounts for the ethnic differences in incidence (24). Polycomb proteins such as RING1B assist in recruiting EWSR1-FLI1 to critical enhancers, further stabilizing its transcriptional regulatory network (25, 26, 40). It is important to emphasize that EWSR1-FLI1 expression and activity are not homogeneous across cells or within tumors, but rather fluctuate reversibly between a “high-activity” proliferative state and a “low-activity” migratory/invasive state; this plasticity is an intrinsic feature of its oncogenic potential and lays the groundwork for metabolic and immune heterogeneity (27, 28).

### 2.2 Reprogramming of glucose metabolism

Similar to most tumors, Ewing sarcoma exhibits classic aerobic glycolysis (the Warburg effect), preferentially converting glucose to lactate even in the presence of abundant oxygen (29–32). EWSR1-FLI1 upregulates key glycolytic nodes, including hexokinase, glucose transporters, and lactate dehydrogenase A (LDHA), providing tumor cells with carbon skeletons and reducing equivalents required for rapid proliferation; LDHA inhibition reduces glycolytic flux and suppresses tumor growth in multiple preclinical models of Ewing sarcoma (33–35). Dual targeting of energy metabolism by combining 2-deoxyglucose with metformin induces apoptosis and reduces stem-like properties across a panel of Ewing sarcoma cell lines (36). These findings were obtained exclusively in two-dimensional monolayer culture, and we do not regard them as evidence of activity against tumor growth. Solid tumors develop internal hypoxia, nutrient depletion and restricted drug penetration, all of which drive phenotypic adaptation and shift drug sensitivity relative to monolayer culture; the combination has not been evaluated *in vivo* in Ewing sarcoma. Because both agents act on core energy-metabolic machinery that also operates in normal proliferating tissue, the risk of additive adverse effects is a further reason to confine interpretation to the *in vitro* setting. Mitochondrion-directed interventions have separately been proposed as a complementary strategy across tumor types (37). Melatonin has been reported to exert cytotoxicity against Ewing sarcoma cells by inhibiting the Warburg effect (38). One direct consequence of glycolysis is the massive efflux of lactate and protons, leading to microenvironmental acidification, which will be detailed in the immunosuppression section.

### 2.3 Reprogramming of amino acid metabolism

EWSR1-FLI1 positively regulates serine-glycine biosynthesis and exogenous serine uptake, providing precursors for nucleotide synthesis and one-carbon metabolism, thereby rendering the tumor dependent on the serine pathway and creating a potential metabolic vulnerability (34, 35, 39). In parallel, glutamine is converted by glutaminase (GLS) into glutamate and enters the tricarboxylic acid (TCA) cycle, supporting macromolecular synthesis and redox balance, representing another hub of tumor metabolism (41–43). Glutamine entry is itself transporter-dependent, and this is where the Ewing-specific layer lies: EWS-FLI1 binds a regulatory element within SLC1A5 (ASCT2) and positively regulates its expression, and a signature comprising SLC1A5 together with PHGDH, PSAT1, PSPH, SHMT2, MTHFD2 and MTHFD1L stratifies patients with Ewing sarcoma into distinct overall-survival groups (34). Because activated effector T cells depend on the same transporter family for glutamine uptake, tumor-cell overexpression of SLC1A5 supplies a concrete molecular basis for nutrient competition at the tumor–immune interface rather than a generic appeal to “glutamine addiction” (Section 3.4). Tryptophan (Trp) metabolism constitutes a third critical axis: indoleamine 2, 3-dioxygenase 1 (IDO1) and tryptophan 2, 3-dioxygenase (TDO2) catalyze the conversion of tryptophan to kynurenine, both depleting local tryptophan and producing immunomodulatory kynurenine; its immunological consequences will be expanded in Chapter 3. Overall, amino acid metabolic remodeling simultaneously serves tumor biosynthesis and immune evasion.

### 2.4 Reprogramming of lipid metabolism

Lipid metabolism is also markedly altered in Ewing sarcoma. Key enzymes of *de novo* fatty acid synthesis, such as fatty acid synthase (FASN) and acetyl-CoA carboxylase (ACC), are upregulated, providing substrates for membrane synthesis and signaling lipids, while altered regulation of fatty acid oxidation affects energy supply and redox status (44–46). Studies on nutritional and metabolic adaptation further reveal that Ewing sarcoma possesses flexible capabilities for lipid and energy acquisition under different nutrient conditions and can be targeted by metabolic reprogramming-modulating agents (47–49). Beyond *de novo* synthesis, Ewing-specific data define several further layers, which we set out here because this section was previously too brief to support the immunological claims made later. First, membrane-lipid remodeling. The core regulatory circuitry operating under EWS-FLI1 (KLF15, TCF4, NKX2-2) directly co-occupies the promoters of FASN, SCD and ACLY, of the sphingolipid enzymes SPTLC1, DEGS1 and UGCG, and of the glycerophospholipid enzyme LPIN2; silencing any of these factors shifts the measured lipidome, with glycerophospholipid and sphingolipid metabolism the two most strongly affected pathways, and the corresponding enzyme inhibitors are cytotoxic to Ewing sarcoma cells (202). Lipid metabolism in this disease is therefore not a passive anabolic requirement but a transcriptionally specified programme. Second, glycerophospholipid biosynthesis as a resistance node: loss of SLFN11 redirects flux toward glycerophospholipid synthesis and drives resistance to DNA-damaging agents, and co-targeting DNA damage together with lipid biosynthesis produces synergistic antitumor effects, identifying a metabolic vulnerability that is created rather than merely accompanied by chemoresistance (203). Third, lipid storage and uptake. Triglyceride esterification and lipid-droplet turnover determine how much free polyunsaturated fatty acid is available for membrane incorporation, and therefore set the threshold at which lipid peroxidation becomes lethal; in Ewing sarcoma this threshold is actively defended, for example through SETD8-dependent signaling, whose loss sensitizes cells to both apoptosis and ferroptosis (204). Fourth, and connecting these to immunity, the lipid species that sustain tumor membranes are the same species taken up by infiltrating CD8+ T cells through CD36, where they drive peroxidation and ferroptotic loss of effector function (Section 3.5). The asymmetry is the point: the tumor invests in ferroptosis defense while the effector compartment does not. Lipid mediators (e.g., prostaglandin E2) and the transfer of tumor-derived lipids to immune cells constitute the material basis through which lipid metabolism influences immunity (50, 51)(**Figure 1**; **Table 1**).

**Figure 1:**
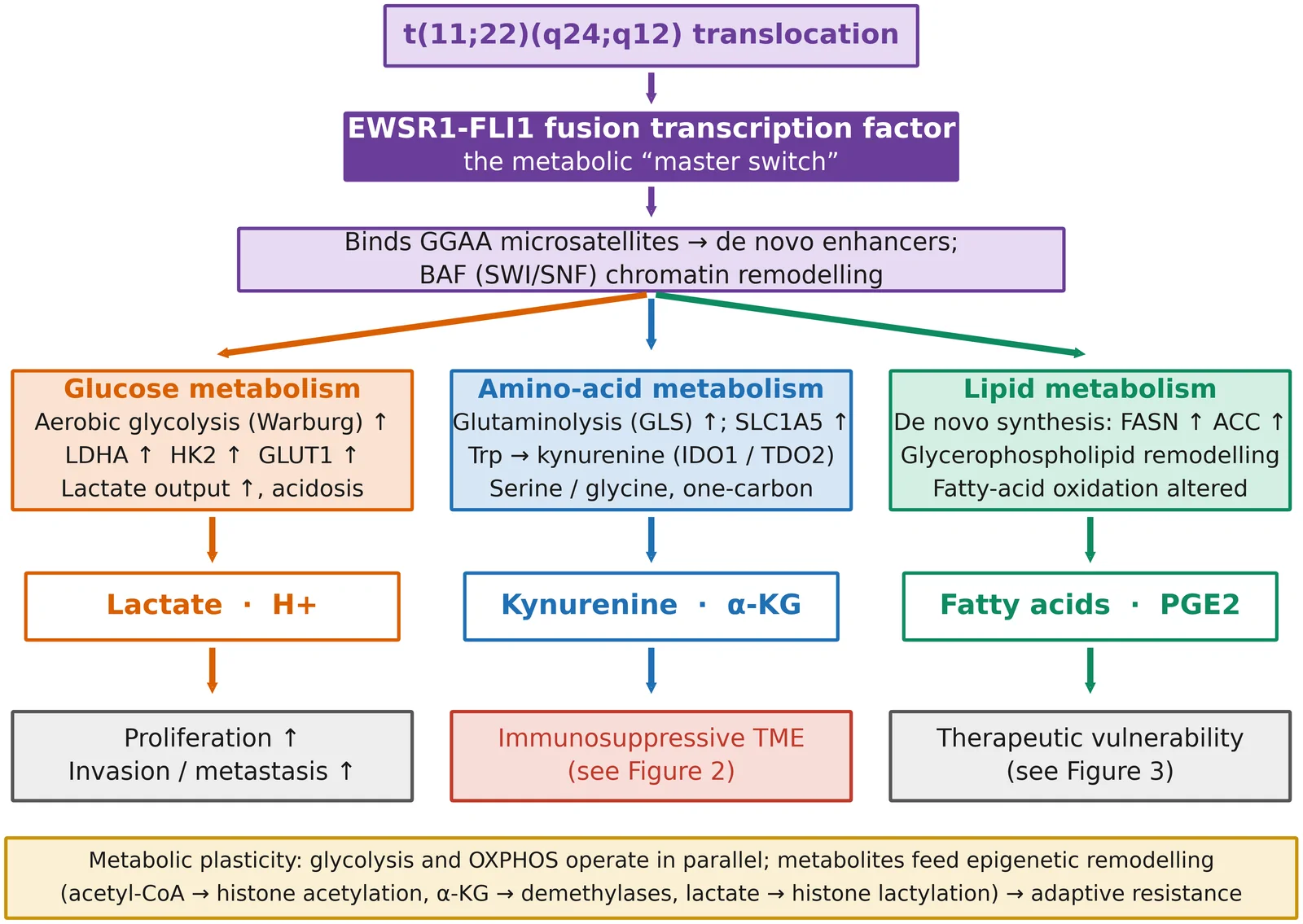
EWSR1-FLI1-driven metabolic reprogramming in Ewing sarcoma. The t(11;22) translocation generates the EWSR1-FLI1 fusion transcription factor, which binds GGAA microsatellites and remodels chromatin to act as a metabolic master switch, coordinately reprogramming glucose, amino-acid, and lipid metabolism and thereby fueling proliferation, invasion, and an immunosuppressive microenvironment. The metabolic-plasticity band is shown below the outcome row so that no connector crosses it. Superscript charges are rendered as plain characters (H+) to avoid glyph substitution, arrow weight has been increased, and font sizes enlarged. ACC, acetyl-CoA carboxylase; BAF, BRG1/BRM-associated factor (SWI/SNF) chromatin-remodeling complex; FASN, fatty-acid synthase; GGAA, (GGAA)n microsatellite; GLS, glutaminase; GLUT1, glucose transporter 1; HK2, hexokinase 2; IDO1, indoleamine 2, 3-dioxygenase 1; α-KG, α-ketoglutarate; LDHA, lactate dehydrogenase A; OXPHOS, oxidative phosphorylation; PGE2, prostaglandin E2; SLC1A5, solute carrier family 1 member 5 (ASCT2); TDO2, tryptophan 2, 3-dioxygenase 2; TME, tumor microenvironment; Trp, tryptophan.

**Table 1:** Key metabolic reprogramming events driven by EWSR1-FLI1 in Ewing sarcoma.

Pathway	Key change	Representative nodes	Functional consequence
Glycolysis	Aerobic glycolysis ↑	LDHA, HK2, GLUT1	Lactate/acidosis; biosynthetic precursors
Glutamine	Glutaminolysis ↑	GLS, glutamine import; SLC1A5 (ASCT2)	TCA anaplerosis; redox balance
Serine/1-C	Serine synthesis ↑	PHGDH, SHMT	Nucleotide & methyl-donor supply
Tryptophan	Kynurenine pathway ↑ (Trp, tryptophan)	IDO1, TDO2	Immunomodulatory kynurenine
Lipid	*De novo* synthesis ↑; glycerophospholipid and sphingolipid remodelling ↑	FASN, ACC; SCD, ACLY, LPIN2, SPTLC1	Membranes; signaling lipids

### 2.5 Metabolic plasticity and epigenetic–metabolic coupling

Metabolism in Ewing sarcoma is not unidirectionally locked into glycolysis; rather, it retains plasticity that allows parallel operation of glycolysis and oxidative phosphorylation, enabling flexible switching under nutritional fluctuation and therapeutic pressure, and there exists a “programmed” nutrient allocation between the tumor and its microenvironment (52). More importantly, metabolic intermediates directly feed back into the epigenetic layer: acetyl-CoA supplies histone acetylation, α-ketoglutarate modulates demethylase activity, and lactate alters gene expression through histone lactylation (see Section 3.2 for details). Acetylation deserves particular emphasis in this disease, since it is the modification most tightly bound to the fusion itself. The *de novo* enhancers that EWSR1-FLI1 establishes at GGAA microsatellites are defined by high H3K27 acetylation, and this enhancer signature is one of the features that most sharply distinguishes Ewing sarcoma from other small round cell tumors (23). The acetyl-CoA pool that supplies H3K27ac is generated by glycolytic and lipogenic flux through ACLY, ACC and FASN (Sections 2.2 and 2.4), which places nutrient availability upstream of fusion-driven enhancer activity rather than only downstream of it. This yields a testable prediction: metabolic restriction that lowers the nucleocytosolic acetyl-CoA pool should attenuate H3K27ac at GGAA enhancers and soften the EWSR1-FLI1 transcriptional output, moving cells toward the low-activity state. We present this explicitly as a hypothesis, because acetyl-CoA-dependent modulation of GGAA-enhancer acetylation has not been directly demonstrated in Ewing sarcoma; if it holds, it would mean that metabolic intervention and epigenetic intervention are acting on the same node from opposite directions. This metabolic–epigenetic coupling enables the tumor to “write” nutritional status into transcriptional programs, and it interweaves with the switching between high- and low-activity states of EWSR1-FLI1, forming a common basis for adaptive resistance and immune heterogeneity (27, 28).

### 2.6 Critical summary: re-examining the causality of the “master switch”

The “master switch” is a useful narrative, but it may imply a linear, homogeneous causality that is not yet supported by the data. At least three caveats should be noted. First, most of the causal evidence for the “metabolism → immunosuppression” mechanisms cited herein derives from models such as melanoma and colorectal cancer; in Ewing sarcoma, these are mostly correlative or extrapolative. Studies that directly manipulate the chain “EWS-FLI1 → a given metabolite → a given immune cell” within Ewing sarcoma models remain few (common mechanistic parallels have been elucidated in other tumor systems, as detailed in Chapter 3). Second, EWS-FLI1 activity is not constant but fluctuates between proliferative and invasive states (27, 28); therefore, the notion that “the fusion gene uniformly drives glycolysis” cannot hold neatly. Indeed, spatial data from paired biopsies in SARC028 show that the immune-evasive phenotype is specifically enriched in tumor subpopulations with high EWSR1-FLI1 target gene expression and low HLA-I (SARC028 spatial data, see Section 5.1.1)—in other words, the immune-metabolic phenotype is “activity-state-dependent” rather than “fusion-constant.” Third, the relationship between hypoxia and EWSR1-FLI1 is bidirectional, and we have taken care to keep this interpretation consistent throughout the review. In one direction hypoxia acts upstream of the fusion: hypoxia induces HIF-1α-dependent accumulation of EWS-FLI1 protein post-transcriptionally, modifies its transcriptional signature, and enhances the malignant properties of Ewing sarcoma cells *in vitro* (205). In the other direction, single-cell analysis of primary tumors finds hypoxia signatures and HIF-1α target genes enriched in subpopulations at both extremes of fusion activity, and the authors of that study stated explicitly that the causal direction could not be resolved from their data (28). We therefore do not describe hypoxia as a downstream output of the fusion. It is better understood as a microenvironmental input that feeds back onto fusion protein level, with the resulting phenotype determined jointly by local oxygen tension and the cell’s starting activity state; where earlier drafts implied a one-way relationship, the text has been corrected. Accordingly, the fusion gene should be better understood as a “necessary scaffold” that sets the stage, whereas which immune-metabolic behavior a given cell ultimately exhibits may depend on its current activity state—a level of nuance that the “switch” metaphor tends to obscure.

## 3 Metabolic reprogramming shapes the immunosuppressive microenvironment

### 3.1 Overall characteristics of the immune microenvironment

Immunohistochemical and transcriptomic studies have shown that PD-1/PD-L1 expression and CD8^+^ infiltration are generally low in Ewing sarcoma (53). Single-cell and spatial transcriptomics have further delineated its immune microenvironment: infiltration is dominated by immunosuppressive myeloid cells, accompanied by functionally impaired lymphocytes and dendritic cells with insufficient antigen-presenting capacity (54). Integrative analyses have identified the MIF-CD74 axis, which simultaneously promotes M2 polarization of macrophages and inhibits CD8^+^ T-cell infiltration; blockade of MIF can “convert cold tumors to hot” and suppress tumor growth (55). Multiple lines of evidence consistently indicate that T cells are scarce, whereas myeloid cells predominate in Ewing sarcoma, and a higher proportion of M2 macrophages correlates with worse prognosis (11, 56, 57). The bone and bone-marrow niche adds a layer that a generic account of the tumor microenvironment omits, and it is worth stating separately because most of the metabolic reasoning in this review is otherwise transferred from soft-tissue tumors. Ewing sarcoma arises in a compartment already occupied by mesenchymal stromal cells, osteoblasts, osteoclasts and marrow adipocytes, each metabolically active and each exchanging substrate with the tumor. Marrow adipocytes constitute a local lipid reservoir; in other marrow-resident malignancies they transfer free fatty acids to tumor cells and support fatty-acid oxidation, which is the same programme on which Tregs and M2 macrophages depend (Section 3.5), so lipid availability in bone is supplied by the niche and not only by tumor lipogenesis. Osteoclast activity driven through RANK/RANKL resorbs mineral and releases protons and sequestered growth factors, which means the acid load in a bone-resident lesion is not attributable to tumor lactate efflux alone and may not respond proportionally to glycolytic inhibition. Osteoblasts and stromal cells compete for the same glucose and glutamine pools as tumor and T cells, making nutrient competition in bone a three-party rather than a two-party problem. Direct measurement of these exchanges in Ewing sarcoma is not yet available, and we raise them not as established mechanism but because they alter the quantitative assumptions behind any metabolic intervention delivered into bone (Sections 5.1.3 and 5.1.4).

### 3.2 Lactate, acidification, and histone lactylation

Lactate and protons generated by glycolysis are core mediators that shape immunosuppression. Classical studies have demonstrated that tumor-derived lactate can suppress human T-cell proliferation and cytokine production by up to approximately 95%, and markedly attenuate cytotoxic activity (58); LDHA-associated lactate production impairs immune surveillance by inhibiting IFN-γ production from T cells and natural killer (NK) cells (59), and polarizes tumor-associated macrophages toward a protumor phenotype (60). Lactate not only acts as an inhibitory signal but also serves as a metabolic substrate upon which regulatory T cells (Tregs) rely for maintenance in a low-glucose, high-lactate environment (61, 62). Reducing tumor lactate (e.g., with dichloroacetate) can restore T-cell function (63). Moreover, histone lactylation is a transcription-activating modification, and we state the direction explicitly because it determines how the mark should be interpreted. Lactate-derived lactyl groups are deposited on histone lysine residues, most characteristically H3K18, at gene promoters, where the mark stimulates transcription of the associated genes rather than repressing them. In macrophages this activating mark accumulates in the later phase of stimulation and directly drives expression of homeostatic, M2-associated genes including Arg1. The immunosuppressive consequence is therefore produced by gene activation, not gene silencing: a glycolytic tumor writes its own metabolic state into an activating chromatin mark that switches the surrounding myeloid compartment into a reparative, protumor programme (64–66).

### 3.3 Tryptophan–Kynurenine–AHR axis

Tryptophan catabolism catalyzed by IDO1/TDO2 produces kynurenine, which serves as an endogenous ligand for the aryl hydrocarbon receptor (AHR) (67–69). Tryptophan depletion suppresses T-cell proliferation through stress-response pathways, whereas kynurenine–AHR signaling promotes Treg differentiation, induces tolerogenic myeloid cells, and upregulates PD-1 expression on CD8^+^ T cells (70–72). In tumors with high IDO/TDO expression, the AHR pathway is active and correlates with immunosuppression; AHR blockade can reverse the Treg–macrophage inhibitory axis (73). Regulation of this axis in Ewing sarcoma is not a simple extrapolation from other tumors, and the direction of the fusion’s effect matters for how the axis should be targeted. In Ewing sarcoma cells, EWS-FLI1 suppresses TDO2: knockdown of the fusion increases TDO2 expression, accelerates tryptophan consumption, and produces accumulation of kynurenine and kynurenic acid, both AHR ligands, with consequent nuclear translocation and activation of AHR; silencing TDO2 reverses both the tryptophan consumption and the kynurenine accumulation (206). Autocrine AHR signaling is thus engaged preferentially in the fusion-low state rather than the fusion-high state. Two limitations were made explicit by the original authors and should be carried forward rather than smoothed over: TDO2 induction after fusion knockdown was observed in only one of five Ewing sarcoma cell lines studied, whereas IDO1 was consistently inducible by IFN-γ across lines, and TDO2 levels in primary tumors vary considerably, correlating with CYP1B1 as a surrogate marker of AHR activation. The practical reading is that IDO1 and TDO2 are likely to dominate in different compartments of the same tumor — IDO1 where interferon signaling is present, TDO2 where fusion activity is low — which is consistent with the activity-state model of Section 2.6 and with the observation that the invasive and hypoxic phenotypes also reside in fusion-low subpopulations. This axis thus provides a well-defined “metabolic–immune” intervention node for Ewing sarcoma, provided that patient and lesion selection accounts for fusion-activity state and for which upstream enzyme is actually operative, rather than treating the tumor as homogeneous.

### 3.4 Glutamine competition and amino acid deprivation

Tumor “addiction” to glutamine depletes glutamine in the microenvironment, thereby constraining T-cell activation and proliferation. Glutamine blockade in tumor-bearing models simultaneously suppresses both oxidative and glycolytic metabolism of the tumor, reducing hypoxia, acidification, and nutrient depletion, while effector T cells shift toward a long-lived, highly active phenotype, revealing an exploitable “metabolic checkpoint” (74–76). Glutaminase inhibitors (e.g., CB-839) can target this dependency (77). Amino acid transport and supply broadly influence T-cell fate (78, 79); arginine, consumed by myeloid cells via arginase, suppresses T-cell receptor expression and antigen-specific responses (80). Nutrient competition thus constitutes an immunosuppressive axis independent of checkpoint pathways (81, 82).

### 3.5 Lipid–immune crosstalk

Lipids play a dual role in immunosuppression. Cholesterol and fatty acids enriched in the microenvironment are taken up by CD8^+^ T cells via transporters such as CD36, inducing lipid peroxidation and ferroptosis, which lead to diminished effector function and exhaustion (83, 84). In contrast, Tregs and M2 macrophages rely on fatty acid oxidation to sustain their suppressive phenotypes (85–88). Myeloid-derived suppressor cells (MDSCs) in tumors shift from glycolysis toward fatty acid oxidation, and enhanced lipid uptake potentiates their immunosuppressive activity (89, 90). Lipid metabolic remodeling therefore creates an asymmetry between effector and suppressor cells: the former are impaired, while the latter benefit. In Ewing sarcoma this asymmetry is compounded by the fact that the tumor cell actively defends its own ferroptosis threshold (Section 2.4) while the infiltrating effector compartment does not. One consequence deserves explicit statement, since it constrains therapeutic design: a strategy that induces ferroptosis in order to kill tumor cells and a strategy that inhibits ferroptosis in order to rescue CD8+ T cells act in opposite directions on the same pathway within the same lesion. Which is preferable is an empirical question about relative thresholds and about whether the two effects can be separated in space or time, and it is not currently answerable from Ewing sarcoma data; we return to it in Section 4.3. Pharmacological interventions targeting metabolic enzymes such as IDO will be discussed in the therapy section (91).

### 3.6 Hypoxia–HIF axis

Because bone is not a normoxic tissue, “hypoxia” requires an explicit operational definition in this setting, and we adopt one here. Ambient air is approximately 21% O2 (about 160 mmHg), whereas physiological oxygen tension in most tissues is 3–7% O2 (roughly 23–50 mmHg), and the bone-marrow niche is lower still, with reported values of approximately 1–6% O2 (about 10–45 mmHg) and the deepest endosteal and perivascular regions at the bottom of that range. Throughout this review, “hypoxia” therefore denotes an oxygen tension below the physiological set-point of the tissue of origin — operationally below 1–2% O2 (under approximately 10–15 mmHg), the range in which HIF-1α escapes prolyl-hydroxylase-mediated degradation — and not simply “below 21% O2”. This distinction is not pedantic: the large majority of *in vitro* studies use ambient air as the normoxic comparator, which systematically overstates the degree of hypoxia relative to the native marrow niche and inflates the apparent magnitude of hypoxia-dependent effects. Rapid proliferation and aberrant vasculature lead to intratumoral hypoxia, which stabilizes HIF-1α and integrates multiple immune evasion mechanisms. HIF-1α directly upregulates PD-L1 on tumor cells and MDSCs, and PD-L1 blockade can enhance MDSC-mediated T-cell activation under hypoxic conditions (92, 93). Hypoxia also impairs immune surveillance by downregulating NK-cell recognition ligands, recruiting Tregs, and attracting suppressive myeloid cells (94–96). Anatomical site is likely to modify this calculus, although it has not been formally tested in Ewing sarcoma. A tumor arising in the diaphysis of a long bone or in the pelvis sits within a marrow compartment whose baseline oxygen tension is already low and whose perfusion is constrained by mineralized cortex, so the increment required to cross a HIF-stabilizing threshold is small and the hypoxic fraction is expected to be large. An extraskeletal soft-tissue primary begins from a higher baseline tension with more compliant vasculature. The therapeutic implications diverge accordingly. Hypoxia-activated prodrugs and HIF-directed agents would be expected to have a wider therapeutic index in skeletal primaries, precisely because the tumor-to-normal oxygen differential there is created by the tumor rather than inherited from the tissue. Strategies that depend on penetration — antibody–drug conjugates, adoptive cell products — face a steeper delivery barrier in the same lesions (Section 5.1.3). Radiotherapy, whose efficacy is oxygen-dependent, is disadvantaged in exactly the skeletal sites where Ewing sarcoma most commonly arises, which is a further argument for combining it with hypoxia-directed rather than oxygen-consuming agents. We propose stratification of metabolic and immune-metabolic interventions by primary site as a hypothesis warranting prospective testing rather than as an established principle.

### 3.7 Overall framework of immunometabolism

Integrating the above mechanisms, metabolic reprogramming and immunosuppression in Ewing sarcoma are not two parallel phenomena but rather two ends of the same causal chain. Immunosuppressive myeloid cells (MDSCs and M2 macrophages) play a pivotal hub role in this process (97–101). Tumor cells and effector immune cells frequently compete for the same nutrients (glucose, glutamine, and arginine), and the tumor typically gains the upper hand in this competition, suppressing mitochondrial biogenesis in infiltrating T cells and resulting in metabolic insufficiency and dysfunction (102–105). For this reason, checkpoint blockade alone—merely “releasing the brakes”—is often insufficient; the “soil” itself must also be remodeled (106–108). Multiple studies suggest that restoring effector immune function may be achievable by limiting glycolysis, modulating Foxp3-dependent Treg metabolism, and intervening in nutrient competition (109–114). This constitutes the logical starting point for the therapeutic strategies discussed below (**Figure 2**; **Table 2**).

**Figure 2:**
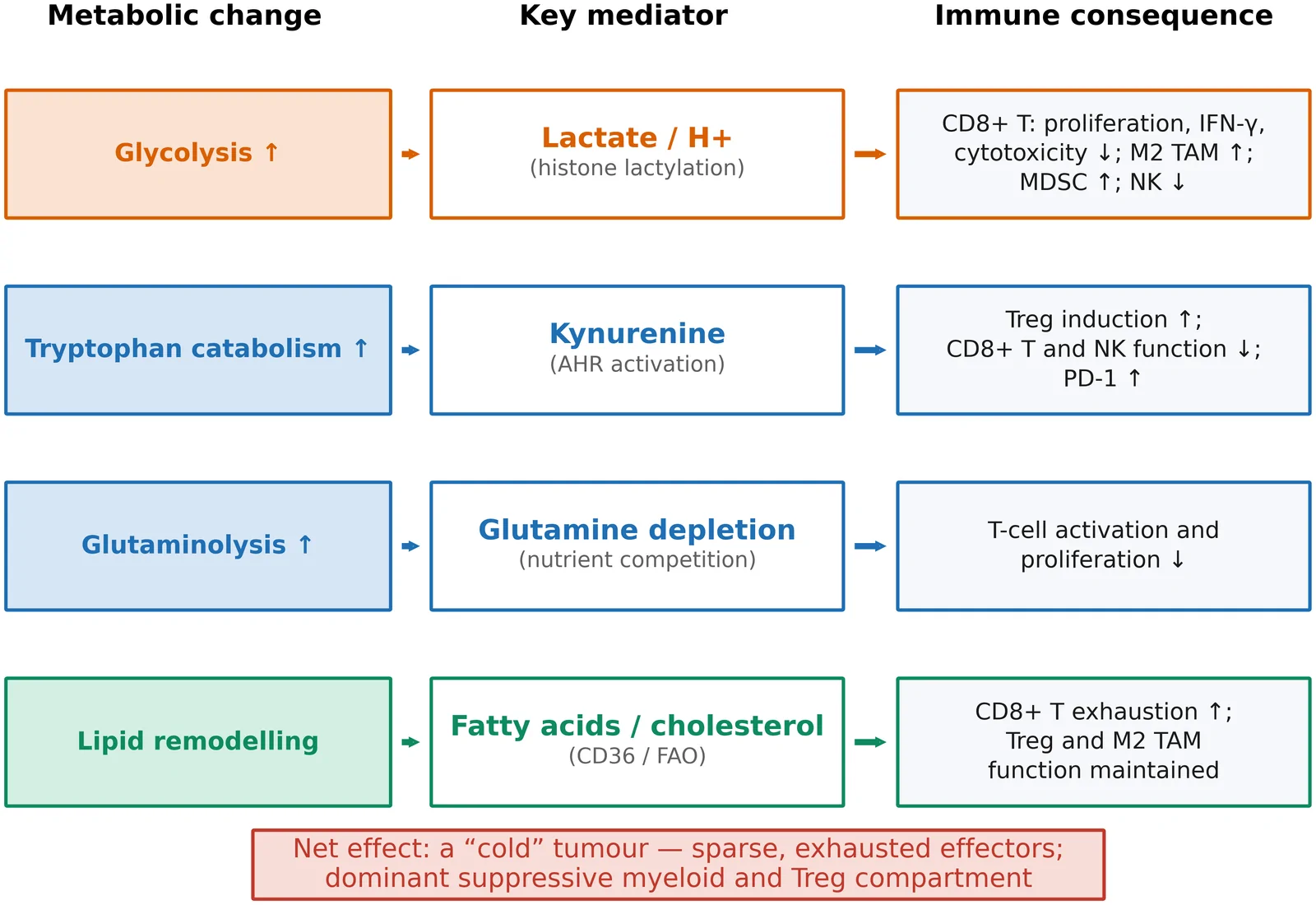
Metabolic reprogramming shapes the immunosuppressive tumor microenvironment. Enhanced glycolysis, tryptophan and glutamine catabolism, and lipid remodeling generate mediators (lactate/H+, kynurenine, glutamine depletion, fatty acids/cholesterol) that impair CD8+ T and NK cells while supporting Treg and M2 macrophage programs, yielding a “cold” tumor. Charges are rendered as plain characters (H+, CD8+), arrows thickened, and labels enlarged. AHR, aryl hydrocarbon receptor; CD36, fatty-acid translocase; FAO, fatty-acid oxidation; IFN-γ, interferon gamma; MDSC, myeloid-derived suppressor cell; NK, natural killer; PD-1, programmed cell death protein 1; TAM, tumor-associated macrophage; Treg, regulatory T cell.

**Table 2:** Metabolite-mediated immunosuppressive mechanisms.

Metabolite/condition	Sensor/effector	Immune target	Net effect	Evidence context
Lactate/H+	MCT; histone lactylation	CD8+ T, NK, TAM	IFN-γ ↓; M2 polarization ↑	Pan-cancer mechanism. Ewing-specific support limited to high glycolytic flux and LDHA dependence (33–35); histone lactylation not yet demonstrated in Ewing sarcoma.
Kynurenine	AHR	Treg, CD8+ T, NK	Treg ↑; PD-1 ↑; cytotoxicity ↓	Directly supported in Ewing sarcoma: EWS-FLI1 represses TDO2, and AHR is activated in fusion-low cells (206). Downstream Treg/PD-1 effects are pan-cancer.
Glutamine depletion	nutrient sensing	Effector T cells	Activation/proliferation ↓	Partly Ewing-specific: EWS-FLI1 upregulates SLC1A5 and glutamine dependence is established (34). T-cell competition inferred from pan-cancer models.
Fatty acids/cholesterol	CD36; FAO	CD8+ T vs Treg/M2	T exhaustion ↑; suppressors sustained	Ewing-specific lipid remodelling and ferroptosis control documented (202–204). CD36-driven CD8+ exhaustion demonstrated only in other tumour types.
Hypoxia	HIF-1α	Tumor & MDSC	PD-L1 ↑; NK recognition ↓	Ewing-specific: hypoxia stabilises EWS-FLI1 protein and hypoxia signatures are enriched in primary tumours (28, 205). HIF-1a-PD-L1 link is pan-cancer.

### 3.8 Critical summary: distinguishing common mechanisms from Ewing sarcoma-specific features

It must be candidly acknowledged that the immune–metabolic mechanisms described in this chapter—lactate and lactylation, the kynurenine–AHR axis, glutamine competition, and cholesterol-driven exhaustion—are nearly all pan-cancer phenomena and have been primarily elucidated outside of Ewing sarcoma. This has two consequences. On the one hand, targeting these common nodes does not confer a therapeutic advantage specific to Ewing sarcoma, but rather inherits the same toxicities and efficacy ceilings already observed in adult trials. On the other hand, the truly distinctive metabolic biology of Ewing sarcoma—the serine/one-carbon and glutamine dependencies imposed by EWS-FLI1 (34, 35, 39, 40), and the fusion-driven “surfaceome” (STEAP1, ENPP1, IL1RAP; see Section 4.4)—has been less frequently integrated into the immune–metabolic narrative. A sharper research agenda should prioritize asking whether the metabolic dependencies unique to Ewing sarcoma create immune vulnerabilities not found in other tumors, rather than repeatedly validating ubiquitous lactate biology in yet another histological type. Equally, the strength of the evidence must be honestly calibrated—the most robust immunological datasets in Ewing sarcoma currently have very small sample sizes (approximately 13 cases in SARC028, only 4 cases in Ewing sarcoma scRNA-seq), are predominantly spatial and descriptive in nature, and the original authors explicitly noted that they did not reach the power for formal statistical comparisons (SARC028 and the osteosarcoma single-cell study, see Sections 5.1.1 and 5.1.3, respectively). Therefore, the “triad” and “ecotype” currently remain robust hypotheses rather than mature biomarkers directly usable for patient stratification.

## 4 Therapeutic strategies targeting the metabolism–immunity interface

### 4.1 Targeting glucose metabolism and acidification

Interventions directed at the glycolysis–lactate–acidification axis carry the potential for a “two-birds-with-one-stone” effect: simultaneously inhibiting tumor growth and improving immune function. Metabolic reprogramming linked to amylin signaling has been shown to induce regression of p53-deficient tumors, suggesting the therapeutic value of this pathway, and targeting lactate metabolism *per se* represents a well-established antitumor strategy (115, 116). Neutralizing tumor acidity can reverse the anergic state of tumor-infiltrating T cells and improve responses to immunotherapy (117, 118). The monocarboxylate transporter 1 (MCT1) inhibitor AZD3965, which blocks lactate transport, has entered early-phase clinical trials (119, 120). The common logic underlying these strategies is to reduce lactate load and alleviate acidification, thereby restoring effector cell function; among these, amylin analogues such as pramlintide inhibit glycolysis in bone and Ewing sarcoma models, providing a rationale for combination with immunotherapy (see Sections 4.4 and 4.5).

### 4.2 Targeting the tryptophan–AHR axis

Although the Phase III ECHO-301 trial of the IDO1 inhibitor epacadostat in combination with pembrolizumab failed to improve outcomes in an unselected melanoma population, this result more likely underscores the need for biomarker-guided patient selection rather than indicating pathway invalidity (121, 122). Direct antagonism of the downstream AHR has emerged as a more attractive alternative: AHR antagonists can reverse the Treg–macrophage inhibitory axis, restore NK-cell recognition, and synergize with checkpoint blockade (123, 124). Other amino acid metabolic nodes, such as methionine, are also expanding the boundaries of “metabolic–epigenetic–immune” interventions (125, 126).

### 4.3 Targeting lipid metabolism

Targeting lipid metabolism aims to both restrict tumor lipid supply and remodel suppressive immune cells. FASN/ACC inhibition can remodel the lipid microenvironment and weaken Tregs and M2 macrophages that depend on fatty acid oxidation (45, 46); restoring lipid homeostasis in CD8+ T cells — by interfering with CD36 or by inhibiting ferroptosis — synergizes with anti-PD-1 to enhance antitumor activity (83, 84). This synergy has been demonstrated in melanoma and other immunologically “hot” models, and we do not claim that it transfers directly to Ewing sarcoma. The objection is well founded: we state in Section 3.1 that PD-1/PD-L1 expression and CD8+ infiltration are low in Ewing sarcoma (53), and a combination whose immunotherapeutic arm depends on releasing a brake that is barely engaged, acting on effector cells that are largely absent, has no coherent rationale as originally written. The claim therefore requires restatement rather than repetition. Lipid-directed intervention in Ewing sarcoma is better framed as a conditioning step intended to make checkpoint blockade meaningful, not as a way of potentiating a response that already exists. If CD36 blockade or ferroptosis inhibition protects the small resident population of CD8+ T cells from lipid-driven exhaustion, and FASN/ACC inhibition simultaneously weakens the Treg and M2 compartment, then the predicted consequence is an increase in effector density and IFN-γ output, which would induce PD-L1 rather than presuppose it. On that logic the defensible sequence is metabolic conditioning first, with checkpoint blockade added only where an on-treatment biopsy demonstrates that effector infiltration and PD-L1 have actually risen — a design that is testable and that differs materially from adding anti-PD-1 to an unselected, PD-L1-low population. An alternative reading, in our view equally reasonable, is that the immunotherapeutic partner in Ewing sarcoma should not be checkpoint blockade at all but antigen-directed cell therapy (Section 4.4), whose activity does not require pre-existing PD-L1. A further caution follows from Section 3.5: because the tumor cell defends its ferroptosis threshold while the effector cell does not, ferroptosis inhibition intended to protect T cells may simultaneously protect tumor cells, and the therapeutic window for that particular manoeuvre remains undefined in this disease. These approaches are mostly in preclinical stages but complement strategies targeting glucose and amino acid metabolism.

### 4.4 Novel surface targets and cell-based therapies

The “surfaceome” of Ewing sarcoma has been systematically mapped in recent years, revealing a panel of surface proteins—including ENPP1 and IL1RAP—that can serve as targets for antibodies, antibody–drug conjugates, or cell-based therapies (127–129). These targets provide leverage for remodeling the myeloid microenvironment beyond direct tumor killing (130, 131). GD2 and B7-H3 (CD276) have shown preliminary efficacy and safety in CAR-T cells for pediatric solid tumors (132, 133), and CAR platforms for sarcoma are being continuously expanded (134, 135). STEAP1 is a direct transcriptional target of EWS-FLI1, induced through binding at GGAA microsatellite elements, and is highly expressed in Ewing sarcoma while remaining largely restricted in normal tissue, which is what makes it tractable where the fusion protein itself is not (136); second-generation STEAP1-BBζ CAR T cells armored with IL-18 produce antitumor activity in orthotopic and disseminated Ewing sarcoma xenograft models at reduced cell doses (137). It is reasonable to ask how such constructs are designed to overcome the specific difficulties of attacking solid tumors, since this is what separates them from the hematological products on which the CAR platform was built, and we set that out briefly here. Three barriers dominate: antigen heterogeneity together with on-target off-tumor toxicity; poor trafficking and physical exclusion by stroma and mineralized matrix; and loss of potency and persistence within a suppressive, nutrient-depleted and acidic microenvironment. Target selection addresses the first — STEAP1 and IL1RAP are chosen precisely because fusion-driven expression is high and comparatively uniform across tumor cells while normal-tissue expression is limited, which narrows rather than eliminates the toxicity window. Cytokine armoring addresses the third, and is the design feature of the construct cited above: locally secreted IL-18 sustains effector function and IFN-γ output *in situ*, permits activity at cell doses an unarmored construct cannot achieve, and repolarizes the surrounding myeloid compartment; systemic recombinant IL-18 was safe but ineffective as monotherapy, so the purpose of armoring is local delivery rather than cytokine exposure *per se*. Costimulatory domain choice is the corresponding persistence lever, with 4-1BB (BBζ) generally preferred over CD28 for slower exhaustion in solid tumors. Beyond these, the wider field is addressing antigen heterogeneity with dual-antigen and logic-gated receptors, poor trafficking with chemokine-receptor engineering and locoregional delivery, and microenvironmental suppression with dominant-negative TGF-β receptors and switch receptors. The design axis most relevant to this review is metabolic fitness: a CAR T cell entering a lactate-rich, acidic, glutamine-depleted lesion encounters exactly the constraints described in Chapter 3, which is the principal argument for the metabolic-conditioning-before-cell-therapy sequence proposed in Section 4.5. We note that none of these constructs has yet been evaluated in a Ewing sarcoma clinical trial. The overall landscape of immunotherapy in Ewing sarcoma—including prior antibody and adoptive cell attempts, as well as genomically guided combination directions—is rapidly evolving (138–145) (**Figure 3**; **Table 3**).

**Figure 3:**
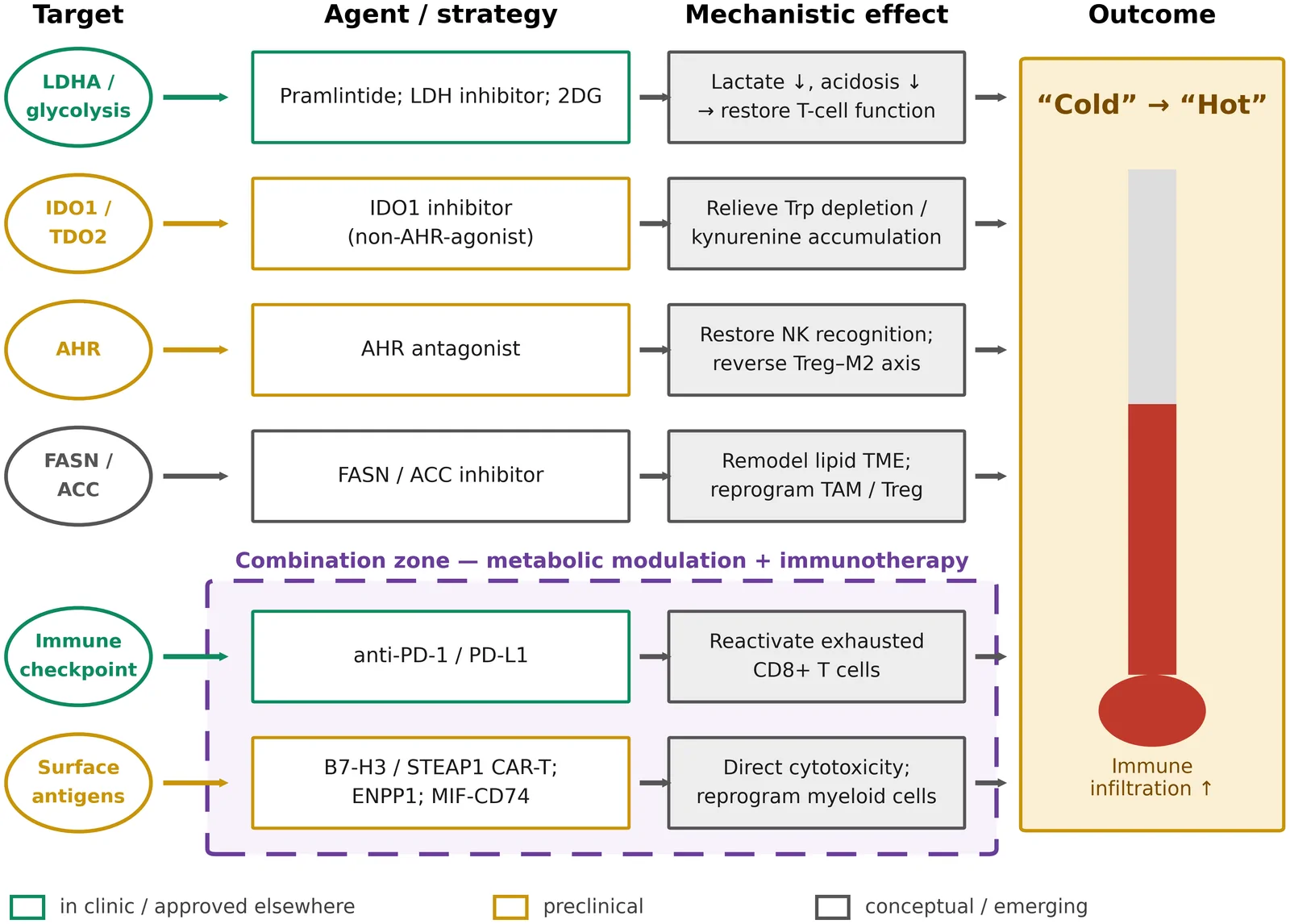
Therapeutic strategies targeting the metabolic–immune interface. Interventions against LDHA/glycolysis, IDO1/TDO2, AHR, FASN/ACC, immune checkpoints, and Ewing surface antigens (B7-H3, STEAP1, ENPP1, MIF-CD74) act through distinct mechanisms to shift the tumor from “cold” toward “hot”. Dashed box indicates the metabolic-modulation-plus-immunotherapy combination zone. Target labels have been moved clear of the node outlines, arrows thickened, and the combination-zone caption repositioned so that it no longer overlies adjacent rows. 2DG, 2-deoxy-D-glucose; ACC, acetyl-CoA carboxylase; AHR, aryl hydrocarbon receptor; CAR-T, chimeric antigen receptor T cell; ENPP1, ectonucleotide pyrophosphatase/phosphodiesterase 1; FASN, fatty-acid synthase; IDO1, indoleamine 2, 3-dioxygenase 1; LDH **(A)**, lactate dehydrogenase **(A)**; MIF, macrophage migration inhibitory factor; NK, natural killer; STEAP1, six-transmembrane epithelial antigen of the prostate 1; TAM, tumor-associated macrophage; TDO2, tryptophan 2, 3-dioxygenase 2; TME, tumor microenvironment; Treg, regulatory T cell; Trp, tryptophan.

**Table 3:** Representative therapeutic strategies at the metabolic–immune interface.

Target	Agent/modality	Rationale	Stage	Evidence context
LDHA/glycolysis	Pramlintide; LDH inhibitor; 2DG	↓ lactate; restore T-cell function	Preclinical	Ewing-specific preclinical: LDHA inhibition active *in vivo* (33). 2DG + metformin is 2D *in vitro* only (36); pramlintide data largely from osteosarcoma (47).
MCT1	AZD3965	Block lactate transport	Phase I	Pan-cancer clinical (phase I). No Ewing sarcoma data.
IDO1	IDO1 inhibitor	Relieve Trp depletion	Phase III (ECHO-301, negative)	Pan-cancer clinical; negative in unselected melanoma (ECHO-301). Not prospectively tested in Ewing sarcoma.
AHR	AHR antagonist	Reverse Treg/M2 axis	Preclinical/early	Pan-cancer preclinical. Ewing-specific rationale from EWS-FLI1-TDO2-AHR regulation (206); no clinical data.
PD-1/PD-L1	Pembrolizumab; nivolumab	Reactivate CD8+ T cells	Phase II (SARC028)	Sarcoma clinical (SARC028). Ewing cohort small and largely unresponsive; PD-L1 generally low.
B7-H3/STEAP1	CAR-T (± IL-18)	Direct cytotoxicity	Preclinical/early clinical	Ewing-specific preclinical for STEAP1 [orthotopic/disseminated xenografts (137)]. B7-H3 and GD2 in early clinical study in other paediatric solid tumours.

### 4.5 Combination strategies: metabolic modulation plus immunotherapy

The central challenge in Ewing sarcoma therapy is how to convert “cold” tumors to “hot.” A rational combination logic is to first “condition the soil” with metabolic/myeloid modulation (reducing lactate acidification, relieving nutrient deprivation, depleting or reprogramming suppressive myeloid cells, and blocking the kynurenine–AHR axis) (146–148), and then “seed” with immunotherapy (checkpoint blockade, CAR-T/CAR-NK, or adoptive NK cells) (149, 150) (**Figure 4**). Multiple studies support the “1 + 1>2” synergy of metabolic modulation combined with immunotherapy (151–153), with mechanisms involving restoration of T-cell mitochondrial function and reversal of Treg stability in glycolysis-deficient tumors (154–156). Combinations of immunotherapy with epigenetic agents (e.g., HDAC, EZH2, and LSD1 inhibitors) are also being explored in pediatric tumors and Ewing sarcoma (157–162). Previous PD-1/PD-L1 monotherapy or limited combination trials in sarcomas (e.g., SARC028, PALETTE, Alliance A091401) have suggested that immunotherapy alone is insufficient, reinforcing the need for combination approaches (163–165).

**Figure 4:**
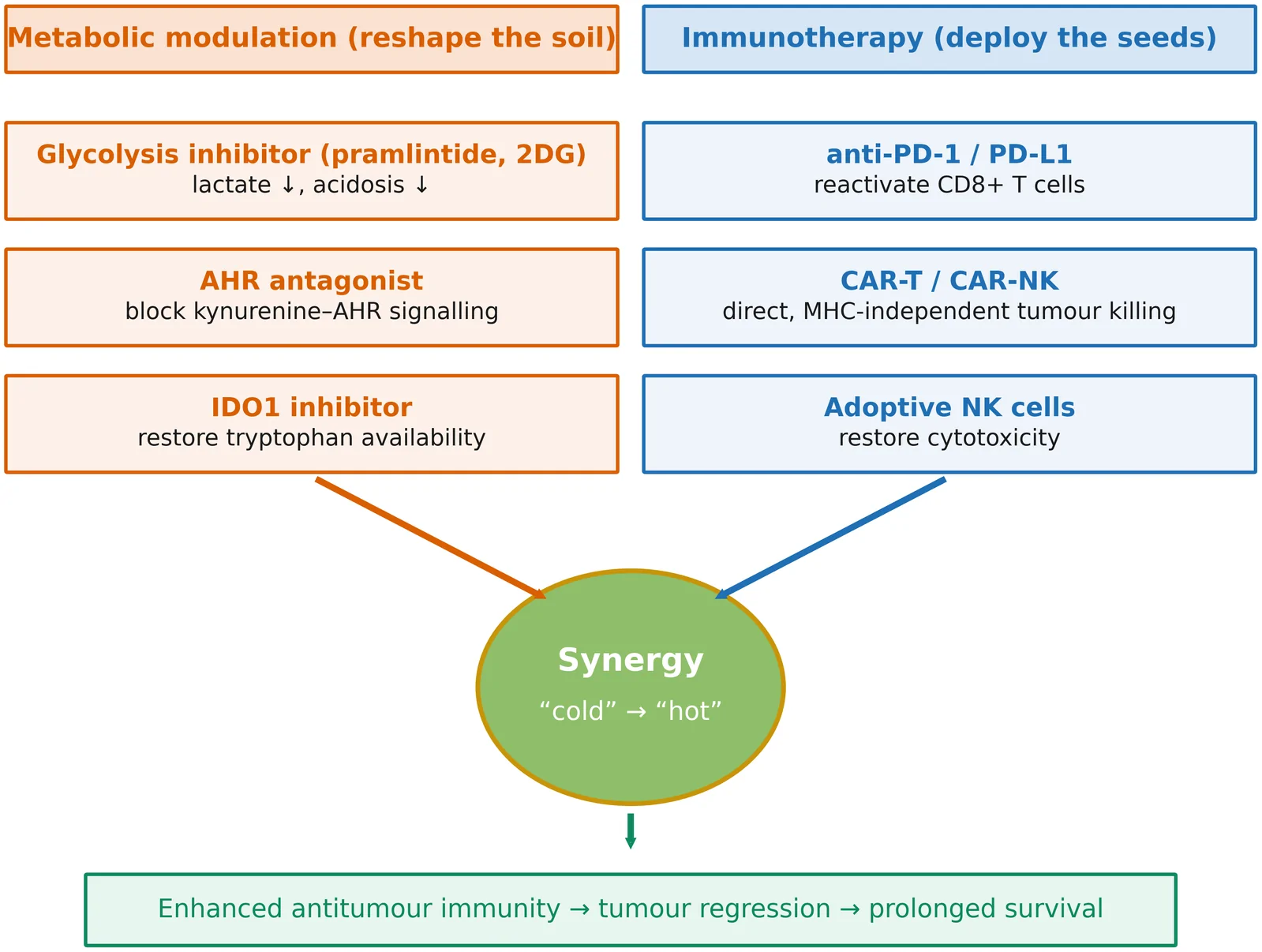
Rationale for combining metabolic modulators with immunotherapy. Reshaping the metabolic “soil” (glycolysis inhibition, AHR antagonism, IDO1 inhibition) together with immunotherapeutic “seeds” (anti-PD-1/PD-L1, CAR-T/CAR-NK, adoptive NK) converges on synergy, converting a cold tumor to hot and enhancing antitumor immunity. The rationale depicted is preclinical, and no combination shown has been validated in a Ewing sarcoma trial. CD8+ is rendered as a plain character and label sizes increased. 2DG, 2-deoxy-D-glucose; AHR, aryl hydrocarbon receptor; CAR-NK, chimeric antigen receptor natural killer cell; CAR-T, chimeric antigen receptor T cell; IDO1, indoleamine 2, 3-dioxygenase 1; MHC, major histocompatibility complex; NK, natural killer; PD-1/PD-L1, programmed cell death protein 1 and its ligand; TME, tumor microenvironment.

### 4.6 Critical summary: boundaries of therapeutic optimism

Each class of strategy carries an often-overlooked ceiling that warrants explicit acknowledgment. The evidence for pramlintide in Ewing sarcoma is actually thin; some data derive from osteosarcoma contexts, and direct extrapolation remains premature (47). AHR is mechanistically the most attractive node, but it is highly pleiotropic—involved in barrier immunity, immune tolerance, and xenobiotic sensing—so the true unknown with systemic antagonism is not whether the target is “rational” but whether a therapeutic window actually exists: excessive blockade may precipitate autoimmunity or barrier toxicity (123, 124). Restraint is also needed at the biomarker level: AHR activity, IDO1, IL1RAP, and others have not been prospectively validated in Ewing sarcoma, and ECHO-301 stands as a cautionary monument in this field—a biologically plausible target with a clearly envisioned biomarker strategy (IDO) that failed in an unselected Phase III population (121, 122). Repeatedly advocating “using metabolic biomarkers to screen patients” in the absence of prospective validation is likely to repeat the same mistake. A more realistic assessment is that the near-term value of metabolic modulation most probably lies as a “preconditioning/adjunct” to immunotherapy, applied to biomarker-defined subgroups, rather than as a monotherapy.

## 5 Major challenges and prospects for clinical translation

### 5.1 Core challenges currently faced

#### 5.1.1 Intrinsic barriers to immunotherapy in Ewing sarcoma

The efficacy of immune checkpoint inhibitors in Ewing sarcoma is extremely limited, with objective response rates for PD-1/PD-L1 monotherapy typically below 10% (166). This does not, however, imply that blockade is entirely without biological effect: spatial proteomic and spatial transcriptomic analyses of paired biopsies from the SARC028 trial revealed that immunomodulatory-related proteins were actually upregulated following pembrolizumab treatment, suggesting a pharmacodynamic response that is simply insufficient to reverse the suppression (166). The true obstacle lies in an intertwined “immunosuppressive triad”—high TGF-β response, low IFN response, and low HLA class I molecule expression. The study identified a CN10 cellular neighborhood that was stably present in progressing patients, in which tumor subpopulations simultaneously exhibited all three features and expressed higher EWSR1-FLI1 target gene programs, thereby linking fusion activity, immunogenicity, and poor prognosis (166). In this context, Ewing sarcoma displays a landscape of sparse T-cell infiltration and dominance of myeloid cells (tumor-associated macrophages and MDSCs) (11, 54, 57). Its low immunogenicity has deeper roots: a relatively “silent” genome, extremely low mutational burden, sparse neoantigens, and limited activation of endogenous retroelements, resulting in an innate deficiency of recognizable targets (16–18, 167, 168). The tumor further actively evades recognition through downregulation of HLA class I molecules (MHC-I), and the status of DNA damage responses additionally modulates the response (169–171). Importantly, these barriers are interrelated and synergistic: low immunogenicity reduces T-cell priming, while myeloid dominance and MHC-I downregulation render residual effector cells both difficult to recognize and actively suppressed, collectively locking Ewing sarcoma into a “cold” and refractory state (172).

#### 5.1.2 Limitations and resistance of metabolic-targeted therapies

Metabolic targeting itself also has notable shortcomings. Glycolysis-modulating agents such as pramlintide can inhibit tumor growth in preclinical models, but monotherapy is rarely curative (47). The core reason is metabolic plasticity: Ewing sarcoma can simultaneously activate glycolysis and mitochondrial respiration, and inhibition of a single pathway is often compensated for by rapid metabolic rewiring, analogous to the selection of resistant clones (32, 51, 52). Metabolic heterogeneity poses another obstacle—different immune cell subsets depend on distinct metabolic pathways: naïve T cells rely on amino acid metabolism to maintain activation potential, whereas NK cells depend on lipid metabolism and the TCA cycle to support their cytotoxic functions (102, 103, 105); thus, a “one-size-fits-all” inhibition of a given metabolic pathway may not only harm effector cells but also fail to suppress suppressor cells. A more practical issue is that the vast majority of metabolic-targeting agents remain at the preclinical stage, very few have entered clinical trials, and the specific pathways underlying acquired resistance remain unclear (79, 88, 173, 174).

#### 5.1.3 Tumor heterogeneity and microenvironmental complexity

Ewing sarcoma presents as “one disease, different faces” at the metabolic and immune levels. Single-cell-based bone tumor atlas data suggest a clear metabolic division of labor among immune subsets: macrophages can differentiate into a lipid-metabolism-driven, immunosuppressive, tissue-repair C06 subset, and a complement-activating, pro-inflammatory C04 subset, with these two “faces” coexisting (175). The direct implication is that broadly targeting the “entire myeloid population” may be counterproductive and requires subset-selective strategies. Combined with evidence on hypoxia and glutamine metabolism, it is likely that Ewing sarcoma harbors more than one metabolic–immune ecotype—one pole characterized by higher immune infiltration with active lipid metabolism, and the other by low infiltration, hypoxia, and glutamine dependence; however, this “typing” remains an emerging hypothesis that awaits validation in Ewing sarcoma-specific cohorts (55, 88, 175). Superimposed on this is the dynamic fluctuation of EWSR1-FLI1 activity: the high-activity state favors proliferation, whereas the low-activity state favors invasion and immune evasion (linked to the tryptophan–kynurenine–AHR axis), rendering therapeutic targets themselves variable over time and space (28). Methylation profiling and immune infiltration subtyping further illustrate significant inter- and intratumoral heterogeneity (176, 177); and the spatial pattern of myeloid dominance and immune exclusion, similarly associated with poor outcomes in adjacent tumors such as osteosarcoma, underscores that understanding heterogeneity is a prerequisite for precision therapy (178, 179).

#### 5.1.4 Institutional and technical bottlenecks in clinical translation

Even when mechanisms are clear, translation remains constrained by multiple practical realities. Ewing sarcoma is a rare disease (annual incidence approximately one to three per million), making patient recruitment difficult and large-scale randomized controlled trials challenging; international multicenter collaboration is almost essential (180, 181). Currently, validated metabolic or immune biomarkers are lacking, leaving patient stratification and response monitoring without a foundation (182, 183). Drug delivery is another hurdle: the mineralized microenvironment and peculiar blood supply of bone tumors limit effective drug access, and bone-associated macrophages along with the RANK/RANKL axis further add complexity to immune regulation (184, 185). The patient population is predominantly children and adolescents, requiring additional consideration of drug safety, long-term toxicity, and effects on growth and development, with more stringent ethical oversight. Moreover, existing cell lines and xenograft models have limited fidelity to human disease, and models that fully recapitulate the human immune microenvironment of Ewing sarcoma are lacking, creating a disconnect between basic and clinical research (186). It is worth noting that “drug repurposing” (e.g., pramlintide) may circumvent some drug development bottlenecks, but still requires resolution of indication definition and dose optimization (47, 115) (**Table 4**).

**Table 4:** Major translational challenges and proposed directions.

Challenge	Description	Proposed direction
Rarity & heterogeneity	Few cases; intra/inter-tumor variability	International networks; metabolic subtyping
Biomarker gap	No metabolic-immune stratifiers	Spatial-omics-guided biomarker discovery
Model limitations	PD-L1 low; poor immune-competent models	Humanized/PDX & organoid platforms
Bone niche	Mineralized barrier; RANK/RANKL	Nanodelivery; niche-targeted approaches

### 5.2 Future prospects for clinical translation

#### 5.2.1 Precision medicine: from “one-size-fits-all” to metabolic subtyping

The first pathway to break through the bottlenecks is “stratified therapy.” If patients could be stratified according to their metabolic–immune status—for example, distinguishing a higher-immune-infiltration/lipid-metabolic type from a low-infiltration/hypoxia–glutamine type—it would be possible to advance treatment from histological classification to molecular–metabolic subtyping (175, 187). Within this framework, predictive biomarkers (e.g., AHR activity, IDO1 expression, IL1RAP levels) and prognostic biomarkers (immune infiltration features, metabolic enzyme expression profiles) could jointly guide regimen selection (55, 127, 128). Dynamic monitoring of tumor metabolic status via liquid biopsy (ctDNA, metabolites, exosomes) may enable real-time adjustment of treatment plans, transforming static “one-time subtyping” into an iteratively updated “metabolic atlas” navigation.

#### 5.2.2 Combination therapy: synergy between metabolic modulators and immunotherapy

The second, and the most central, pathway of this review is to employ metabolic intervention as the key to unlocking immunotherapy in Ewing sarcoma. The theoretical basis is that metabolic modulation can reduce tumor acidification and remodel the metabolic microenvironment, thereby enhancing immune cell infiltration and function, driving the conversion from “cold” to “hot, “ and complementing immunotherapy mechanistically (115, 117, 118). In concrete terms: glycolysis targeting (e.g., pramlintide) reduces lactate and acidification and can be combined with PD-1/PD-L1 inhibitors to improve T-cell migration and function (47, 119, 120); AHR antagonists lie at the intersection of “fusion activity fluctuation – amino acid metabolism – immune evasion, “ and their combination with checkpoint inhibitors represents one of the most promising pairings, while caution should be exercised with IDO1 inhibitors to select non-AHR-agonistic compounds so as not to offset the benefit (121–124). The “1 + 1 > 2” rationale for such “metabolic + immune” combinations is supported by extensive preclinical and translational evidence, with mechanisms involving restoration of T-cell mitochondrial function and reversal of Treg stability in glycolysis-deficient tumors (149–156). Early attempts at myeloid-targeting (anti-CSF1R) or anti-angiogenic combinations with immunotherapy in sarcoma also provide reference points for combination design (188, 189). The optimal timing, dose, and sequence of administration should be investigated as key parameters in preclinical studies, and validation should begin with biomarker-screened Phase Ib/II umbrella trials.

#### 5.2.3 Emerging technologies and novel drug development

The third pathway derives from continuous iteration of technologies and new agents, following the logic of “discover new targets → validate in new models → develop new drugs → enter new trials.” Single-cell and spatial omics are revealing, at unprecedented resolution, the metabolic plasticity and spatiotemporal heterogeneity of immune cells, laying the foundation for target discovery (54, 175); dual-dimensional analysis combining surface proteomics with global proteomics has identified novel immunotherapeutic targets such as ENPP1 and IL1RAP (127, 128). Nano-delivery systems hold promise for overcoming drug delivery hurdles in bone tumors (185); tumor organoids and humanized mouse models can accelerate drug screening and optimization/validation of “metabolic–immune” combination regimens. In terms of therapeutic modalities, antibody–drug conjugates represented by surfaceome targets such as B7-H3, as well as CAR-T/TCR-T and CAR-NK cell therapies, are rapidly evolving; allogeneic “off-the-shelf” products and CAR-macrophages further broaden the boundaries of cell therapy for solid tumors (132, 133, 136, 137, 190–192). Epigenetic–metabolic combinations (e.g., targeting EZH2, HDAC, or LSD1 with metabolic intervention) and novel small molecules for Ewing sarcoma (including relevant Phase II investigational agents) are also continuously replenishing the therapeutic arsenal (160–162, 193).

#### 5.2.4 Translational roadmap from bench to bedside

Synthesizing the above, we propose a phased roadmap (**Figure 5**). Short-term (1–3 years): complete additional preclinical validation of metabolic-targeting agents, especially AHR antagonists; initiate Phase I/Ib trials of metabolic modulators combined with immunotherapy; establish biomarker-based patient stratification protocols; and refine humanized animal models for Ewing sarcoma (194). Medium-term (3–5 years): validate combination efficacy in Phase II trials in relapsed/refractory populations; advance biomarker-guided precision strategies and clinical studies of novel immunotherapies (CAR-T, antibody-drug conjugates); and establish and validate a metabolic subtyping system (195, 196). Long-term (5–10 years): achieve individualized treatment based on metabolic–immune subtyping; move effective combinations to the frontline setting; strive to elevate the long-term survival of metastatic patients from approximately 20% to above 50%; and leverage international rare sarcoma collaborative networks to pool samples and data (197–199). Throughout, the overarching principle is to recognize and intervene in metabolism and immunity as a coupled system, and through fine-tuned regulation of the myeloid and bone microenvironments, gradually convert “cold” tumors to “hot” (200, 201).

**Figure 5:**
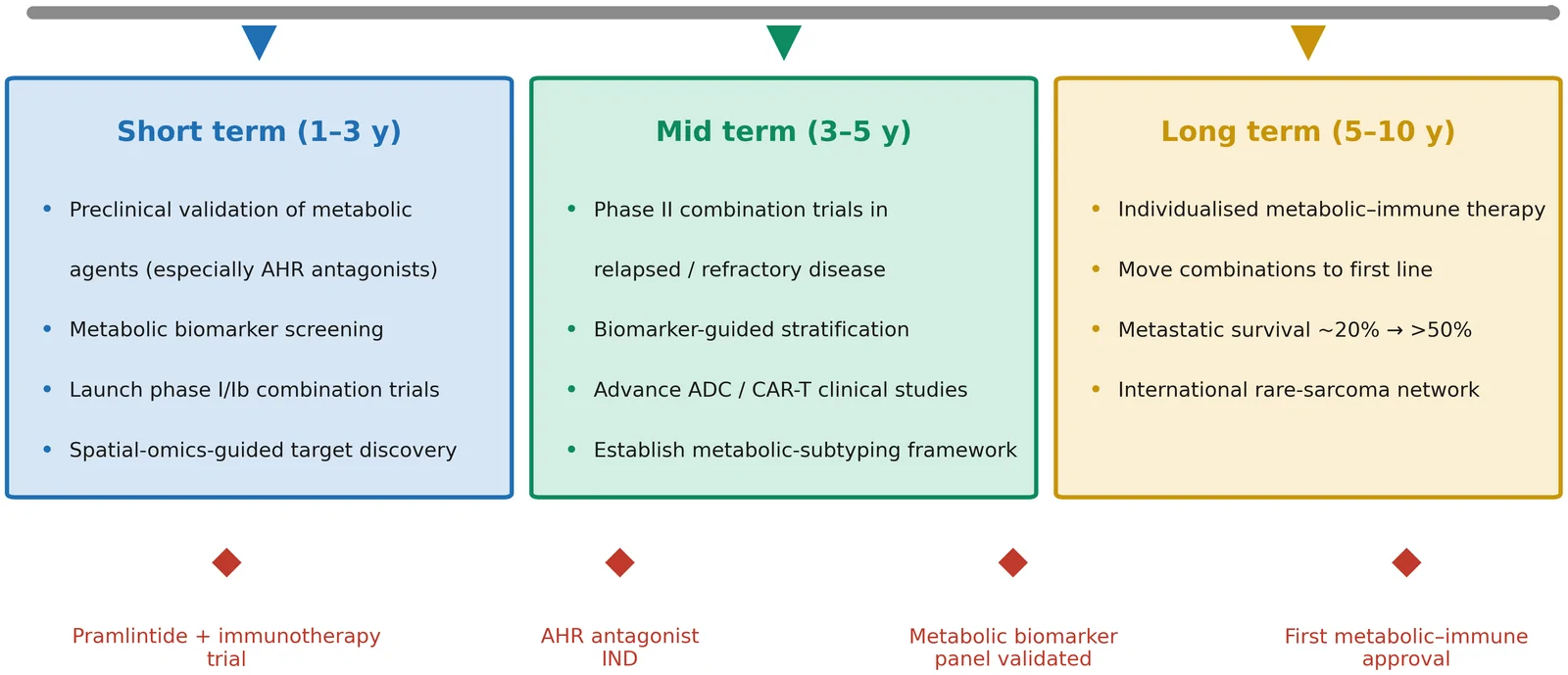
Bench-to-bedside translational roadmap. A staged plan spanning short- (1–3 y), mid- (3–5 y), and long-term (5–10 y) milestones, from preclinical validation and biomarker discovery to biomarker-guided combination trials and individualized metabolic–immune therapy. The timeline has been widened and all text enlarged; milestone markers use plain geometric glyphs in place of the symbol font that previously failed to render. ADC, antibody–drug conjugate; AHR, aryl hydrocarbon receptor; CAR-T, chimeric antigen receptor T cell; IND, investigational new drug application.

## 6 Integrative perspective: a metabolic-immune coupling model based on the activity state of EWS-FLI1

### 6.1 The model: two interconvertible coupled modules defined by activity state

Synthesizing the foregoing evidence, we propose a testable integrative framework: conceptualize the immunosuppression in Ewing sarcoma as two coupled modules defined by EWS-FLI1 activity states, which are interconvertible. The high-activity state (high EWS: FLI1 target gene expression, strong glycolysis, low HLA-I and IFN response) constitutes an antigen-poor, immunosuppressive “tumor core, “ aligning with the c1/c4 subpopulations enriched in CN10 and associated with worse prognosis in the SARC028 spatial data (166). The low-activity state (hypoxia, invasion/migration, stromal remodeling) drives dissemination and likely achieves immune evasion through a distinct hypoxia–AHR-related program, echoing single-cell observations where high activity accompanies hypoxia and low activity accompanies invasion (27, 28). The plasticity and reversibility of these two states precisely explain two long-standing puzzles: why inhibition of a single metabolic node often fails—the surviving alternate state compensates—and why the “cold” immune status is so tenacious—each state carries its own intrinsic escape mechanisms.

### 6.2 Therapeutic corollary: from empirical add-on to state-guided combination

If this model holds, “combination” should no longer be an empirical add-on but rather a design tailored to states. Durable benefit likely requires simultaneous pressure on both states: restoring antigen presentation and HLA-I in the high-activity core (via epigenetic de-repression or IFN pathway activation) (160–162), dismantling the hypoxia–AHR program at the low-activity front (123, 124), and superimposing immunotherapy to harvest the resulting recognizability (149–151). In other words, the role of metabolic intervention is not “direct killing” but “releasing the respective immune brakes of both states, “ creating conditions for immunotherapy that it cannot achieve alone; this also explains why combinations centered on AHR antagonists are most attractive—AHR sits precisely at the intersection of the low-activity, invasive state and immune evasion.

### 6.3 Strength of evidence and falsifiability

It must be emphasized that the above model is currently built upon small-sample, predominantly correlative human data and awaits prospective validation; it is entirely falsifiable. The experiments that would truly test it should: in paired (pre- vs. post-treatment) samples, simultaneously resolve EWS-FLI1 activity, metabolic flux, and HLA-I/IFN status at single-cell/neighborhood resolution to test whether “high activity – low antigen – immunosuppression” stably co-localizes (166, 175); and, using lineage tracing *in vivo*, observe whether cells escape through activity-state switching when subjected to simultaneous metabolic and immune pressure (28). If the two states are not causally linked to their respective immune programs, or if pressure on a single state alone is sufficient to durably convert the tumor to “hot, “ the model would be weakened. Stating these criteria explicitly is itself what distinguishes this framework from a generic review.

### 6.4 Authors’ perspective

Based on the available evidence, our judgment is that metabolic reprogramming is more likely an amplifier and maintainer of the “cold” immune state in Ewing sarcoma, rather than its sole initiating cause; the “antigen-side” defects—low mutational burden and HLA-I deficiency—are probably more upstream constraints (16–18). Therefore, modulating metabolism alone without repairing antigen presentation is unlikely to generate a durable immune response in Ewing sarcoma. The most promising path is to combine “antigen-side restoration + state-guided metabolic unlocking + immunotherapy, “ and to select patients using truly prospectively validated biomarkers—this is both our core proposal and a necessary, evidence-based tempering of the prevailing optimism.

## 7 Conclusion

The therapeutic dilemma of Ewing sarcoma is rooted in its “cold” and suppressive immune microenvironment, which is largely a product of EWSR1-FLI1-driven metabolic reprogramming. The glycolysis–lactate axis, the tryptophan–kynurenine–AHR axis, glutamine competition, lipid–immune crosstalk, and the hypoxia–HIF axis collectively translate metabolic status into immunosuppression. Intervening in metabolism and immunity as an integrated coupled system—first remodeling the metabolic “soil” and then unleashing the immune “seeds”—holds the greatest promise for breaking through the current therapeutic bottleneck. Combination strategies grounded in metabolic subtyping, aimed at “converting cold to hot, “ and guided by biomarkers warrant testing in well-designed early-phase clinical trials.
